# Rupture of intrahepatic bile duct after ERCP for common bile duct stone: A case report of rare complications

**DOI:** 10.1097/MD.0000000000039283

**Published:** 2024-08-16

**Authors:** Tuan Huu Ly, Quang Tien Pham, Loc Huynh Tran, Ha Nhat Tran, Phuoc Duy Tran, Khoa Hoang Anh Nguyen, Truc Huynh Thanh Le, Trung Quoc Pham

**Affiliations:** a Department of Surgery, Faculty of Medicine, University of Medicine and Pharmacy at Ho Chi Minh City, Vietnam; b Department of Surgery, Nhan Dan Gia Dinh Hospital, Ho Chi Minh City, Vietnam; c Department of Surgery, Pham Ngoc Thach University, Vietnam.

**Keywords:** bile duct injury, complication, ERCP, intraoperative cholangiogram

## Abstract

**Rationale:**

Complications after endoscopic retrograde cholangiopancreatography (ERCP) are diverse and usually treated with nonoperative management or percutaneous drainage; however, there are still some rare, life-threatening complications. This is an extremely rare case of biliary peritonitis caused by rupture of the intrahepatic bile duct after ERCP.

**Patient concerns:**

A 63-year-old male underwent ERCP for common bile duct stones. On the second day after the procedure, the patient developed sepsis and abdominal distention. Contrast-enhanced computed tomography revealed a subcapsular hepatic fluid collection attached to the bile duct of segment VII.

**Diagnoses:**

Sepsis resulted in liver parenchyma rupture and intrahepatic bile duct injury after ERCP. Intraoperative cholangiography revealed a connection between a hole in the liver parenchymal surface and the intrahepatic bile duct.

**Interventions:**

Surgeons performed the cholecystectomy, inserted a T-tube into the common bile duct stones, sutured the defect, and put 2 drainage tubes around the lesion.

**Outcomes:**

Postoperative recovery was uneventful, and the patient was discharged on the 17th postoperative day.

**Lessons:**

Intrahepatic bile duct perforation after ERCP can lead to rupture of the liver parenchyma, biloma, or abdominal peritonitis. Multidisciplinary management is necessary to achieve favorable outcomes.

## 1. Introduction

Endoscopic retrograde cholangiopancreatography (ERCP) has been used as a therapeutic tool for various pancreaticobiliary disorders and diseases. ERCP is a complex procedure that requires specialized training and experience. Although this is a minimally invasive procedure, ERCP may lead to complications such as pancreatitis, bleeding, cholangitis, duodenal perforation etc.^[1]^ Post-ERCP duodenal or biliary perforations are serious complications with an incidence rate of 0.6% and fatality rate of 9.9%.^[2]^ According to Stapfer, post-ERCP perforative complications are classified as duodenal and bile duct injuries, and their mechanisms include sphincterotomy, guidewire, or endoscopy. Liver parenchyma perforation may be related to the guidewire and is an extremely rare complication.^[3,4]^

In our case report, the radiologist believed that post-ERCP biliary peritonitis was caused by an injured segment VIII bile duct. Liver parenchyma perforation was confirmed during surgery by cholangiography and methylene blue injection through the T-tube. The following case report is in line with the SCARE criteria.

## 2. Presentation of case

A 63-year-old male was admitted to our hospital with a 6-month history of epigastric abdominal pain, fever, jaundice, and no weight loss. His medical history included hypertension and surgery for a femoral fracture. Physical examination revealed tenderness on palpation of the right upper quadrant, without rebound or guarding.

Laboratory examinations revealed normal results, but magnetic resonance imaging showed common bile duct (CBD) stones and dilation of the intrahepatic and extrahepatic bile ducts, with the diameter of the CBD being 9 mm. Furthermore, the clinician detected a gastrointestinal stromal tumor in the stomach via upper gastrointestinal endoscopy, which was 2 centimeters in size. The patient underwent endoscopic retrograde cholangiopancreatography (ERCP) for choledocholithiasis. A widened common bile duct was observed with small stones next to the papilla. A sphincterectomy with balloon and stone removal was performed. No complications were observed.

On postoperative day postoperation, the patient complained of abdominal pain and fever. Physical examination revealed generalized distension, abdominal tenderness, and guarding. His vital signs were high temperature (38°C), tachycardia (110 bpm), and normal blood pressure (120/70 mm Hg).

**Figure 1.**
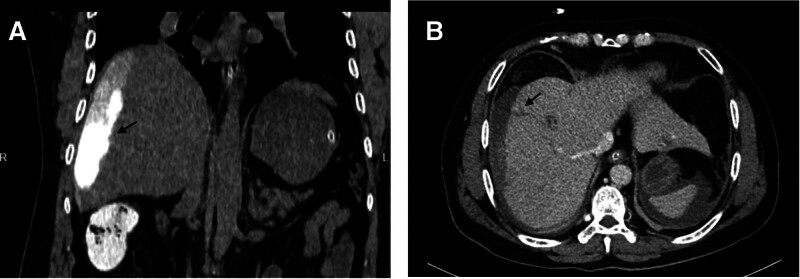
(A) A fluid collection with contrasting agent was seen at the non-contrast phase (arrow), and this contrast agent was suspected the remainder after ERCP. (B) The bile duct of segment VIII was connected with the subcapsular fluid collection (arrow). ERCP = endoscopic retrograde cholangiopancreatography.

Laboratory tests revealed elevations in the white blood cell count (18.13 K/µL) with neutrophil predominance (93.9%), C-reactive protein level (308.6 mg/L), total bilirubin (81.64 µmol/L), direct bilirubin (51.75 µmol/L), and AST/ALT (111/176 U/L). The patient underwent urgent computed tomography (CT) with contrast, which demonstrated strikingly free fluid in the upper peritoneal cavity and a subcapsular hepatic fluid collection attached to the bile duct of segment VIII, which was suspected to be a bile leak (Fig. 1A, B).

**Figure 2.**
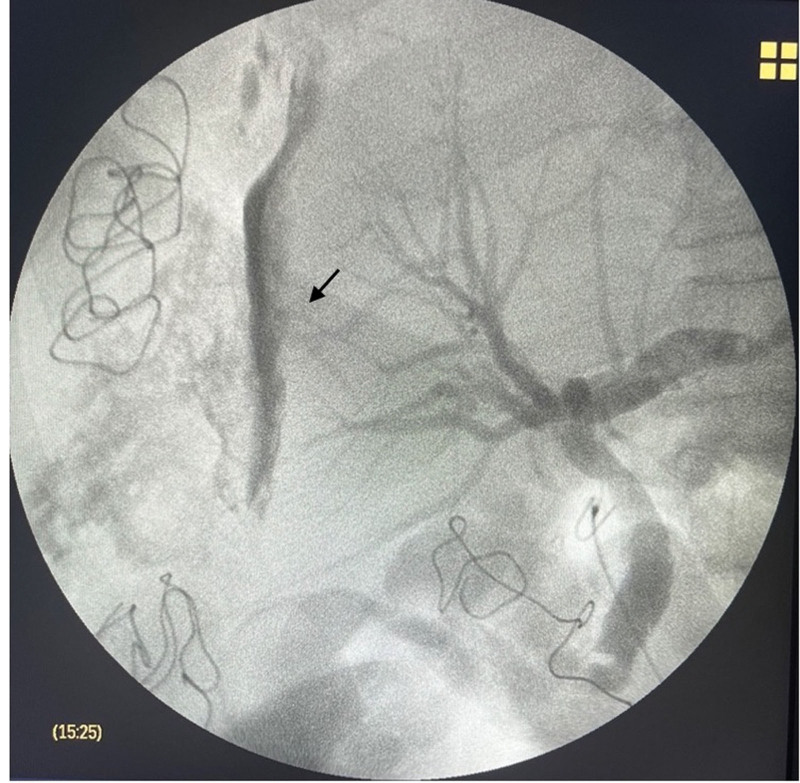
The contrast collection linked to the intrahepatic bile duct (arrow).

The patient was indicated for emergency surgery, with a diagnosis of biliary peritonitis on the second day of ERCP. The most striking intraoperative features were biliary peritonitis and a large subcapsular biloma at hepatic segments VI–VII, which were partly ruptured. The surgeons decided to perform an intraoperative cholangiography through the cystic duct after cholecystectomy. The contrast medium leaked to the periphery of hepatic segments VI–VII (Fig. 2).

**Figure 3.**
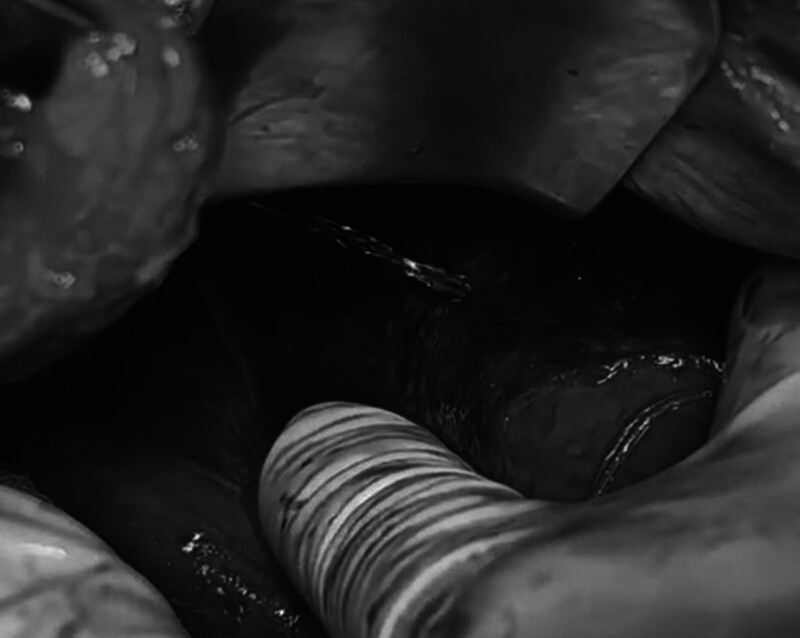
A hole in the surface of the liver resulted from the rupture of the intrahepatic bile duct.

The T-tube drainage was positioned to facilitate biliary drainage, and the biloma was removed. When methylene blue was injected through the T-tube, blue fluid was gushed out through a hole approximately 1 cm in segment VII, with rough liver parenchyma. The defect was closed using 2 U-shape sutures (Fig. 3). Finally, 2 drainage tubes were placed beside the hole to prevent bile leakage.

The patient was referred to the intensive care unit because of sepsis and pulmonary and cardiovascular failures. Fortunately, the postoperative recovery was uneventful, and the organ dysfunction was retreated. A CT scan on postoperative day 11 revealed no free fluid in the peritoneum. Finally, the drainage tubes were removed and the patient was discharged on postoperative day 17.

## 3. Discussion

Although ERCP is accepted for its efficacy and safety in treating pancreaticobiliary diseases, the procedure remains susceptible to common complications, such as pancreatitis, hemorrhage, and cholangitis, as well as rare complications, such as biliary and duodenal perforation.^[5]^ Biloma, identified as an encapsulated extrahepatic or intrahepatic collection of bile fluid, is rare after ERCP. Moreover, rupture of the biloma is extremely rare and could lead to total biliary peritonitis and life-threatening severe sepsis.

Biloma mainly results from iatrogenic injuries such as hepatobiliary surgery, percutaneous transhepatic procedures, hepatobiliary trauma, ERCP-related procedures, and spontaneous rupture of the bile duct.^[5,6]^ Most bilomas may be treated with nonoperative management (NOM) or a combination of percutaneous and endoscopic drainage.^[7–9]^ Similarly, in occasional reports of biloma detected after ERCP, the authors opined about the success of NOM or drainage.^[5,10,11]^

In this case, the combination of clinical signs and CT scan features provided an immediate and accurate diagnosis. During the operation, the main purposes are drainage of the bile duct and washing out the bile fluid intraperitoneally. Through cholangiography, a defect in the liver parenchyma is detected, but suturing the hole in the liver surface is more difficult because of the rough liver parenchyma and lack of the Glisson capsule.

Reviewing the literature, there are some cases of biloma after ERCP that were successfully treated with NOM and drainage,^[5,10–12]^ and there are 2 cases of ERCP-related biliary peritonitis.^[3,4]^

The 2 main hypotheses for intrahepatic biliary injuries after ERCP are as follows: lesions caused by excessive aspiration of the guidewire or inappropriate materials of the guidewire,^[4,13]^ and increasing the intra-bile duct pressure through air insufflation during ERCP.^[3,14]^ Furthermore, edema of the Vater papilla after the procedure could cause an increase in size and pressure in the bile duct tree on postoperative days, leading to rupture of the biloma and contributing to biliary peritonitis.^[5]^ In this case, the hole on the liver surface was large and had a rough edge. Therefore, the explosion of the terminal segmental bile duct by suddenly increasing pressure in the bile duct tree could explain the complex lesions.

In this case, there are many solutions to prevent complications from ERCP, such as skilled and experienced endoscopists, essential instruments, avoiding air over-insufflation into the bile duct, and even replacing air with carbon dioxide in the procedure.^[14]^ Furthermore, it is necessary to cooperate and coordinate among endoscopists, radiologists, and surgeons in the monitoring, examination, diagnosis, and management of post-ERCP patients.

## 4. Conclusion

In conclusion, this was an extremely rare complication of ERCP. A sudden increase in intrabiliary pressure causes rupture of the terminal bile duct, leading to liver parenchymal explosion, biloma, and biliary peritonitis. Multidisciplinary management is necessary in complex situations to obtain good outcomes for patients.

## Acknowledgments

The patient and his next of skin has provided informed consent for publication of this case. The authors declare that they have no affiliations with or involvement in any organization or entity with any financial interest in the subject matter or materials discussed in this manuscript.

The authors identify and correct errors in language and technical compliance by Paperpal Preflight Service.

## Author contributions

**Supervision:** Tuan Huu Ly, Ha Nhat Tran.

**Visualization:** Tuan Huu Ly, Quang Tien Pham, Loc Huynh Tran, Ha Nhat Tran.

**Writing – original draft:** Ha Nhat Tran.

**Writing – review & editing:** Tuan Huu Ly, Quang Tien Pham, Loc Huynh Tran, Ha Nhat Tran, Phuoc Duy Tran, Khoa Hoang Anh Nguyen, Truc Huynh Thanh Le, Trung Quoc Pham.
